# Malignant Prolactinoma With Liver Metastases Masquerading as Metastatic Gastrointestinal Stromal Tumor: A Case Report and Literature Review

**DOI:** 10.3389/fendo.2020.00451

**Published:** 2020-07-14

**Authors:** A Ram Hong, Jee Hee Yoon, Hee Kyung Kim, Ho-Cheol Kang

**Affiliations:** Department of Internal Medicine, Chonnam National University Medical School, Gwangju, South Korea

**Keywords:** prolactinoma, pituitary carcinoma, dopamine agonist, gastrointestinal stromal tumor, metastasis

## Abstract

Pituitary carcinomas are rare diseases defined as pituitary tumors with metastases. In this report, we describe a case of malignant prolactinoma with liver metastases masquerading as metastatic gastrointestinal stromal tumor (GIST). A 54–years–old woman received dopamine agonists for macroprolactinoma for 2 years, followed by transsphenoidal surgery due to a poor response to medical therapy. Despite the continuation of dopamine agonist after surgery, serum prolactin level progressively increased to above 8,000 ng/ml in 5 years. There was no evidence of disease recurrence on sella magnetic resonance imaging (MRI). She stopped medical therapy. Meanwhile, she was diagnosed with GIST accompanied by liver and peritoneal metastases. After a 2–months treatment with imatinib, she suddenly presented with headache and visual impairment. Sella MRI showed a 3.3–cm sized pituitary mass, and serum prolactin levels were still high. For the recurred mass, she underwent a second surgery followed by radiation therapy. During the imatinib treatment for GIST, main mass and peritoneal metastases were dramatically decreased, but liver metastases were markedly aggravated. Liver masses were eventually confirmed as metastases from prolactin-producing pituitary carcinoma and not from GIST by percutaneous biopsy. Unfortunately, she died 6 months after the second surgery due to acute renal failure and sepsis. This case suggests that highly sustained serum prolactin levels during the dopamine agonist may indicate prolactin-producing pituitary carcinomas with hidden metastases.

## Introduction

Malignant prolactinomas are exceedingly rare diseases that are defined as prolactin (PRL)-producing pituitary tumors with cerebrospinal, meningeal, or distant metastasis (1). The incidence of pituitary carcinomas is about 0.1–0.2% of all pituitary tumors, and malignant prolactinomas are the second common subtype of pituitary carcinomas (2). Pituitary carcinomas are indistinguishable from aggressive adenomas unless metastases are detected, because they show similar clinical and histological features. Similar to other neuroendocrine tumors, several pathological markers including mitotic index, Ki-67, and p53 cannot clearly predict the metastatic potential of pituitary tumors. Although benign prolactinomas respond well to medical therapy with dopamine agonists (DAs) including bromocriptine and cabergoline, treatment options for malignant prolactinomas are limited, and the prognosis is usually poor (3).

Here, we present an unusual case of malignant prolactinoma with liver metastases that were mistaken for metastatic lesions of a concurrently occurring gastrointestinal tumor (GIST).

## Case Presentation

**Figure 1:**
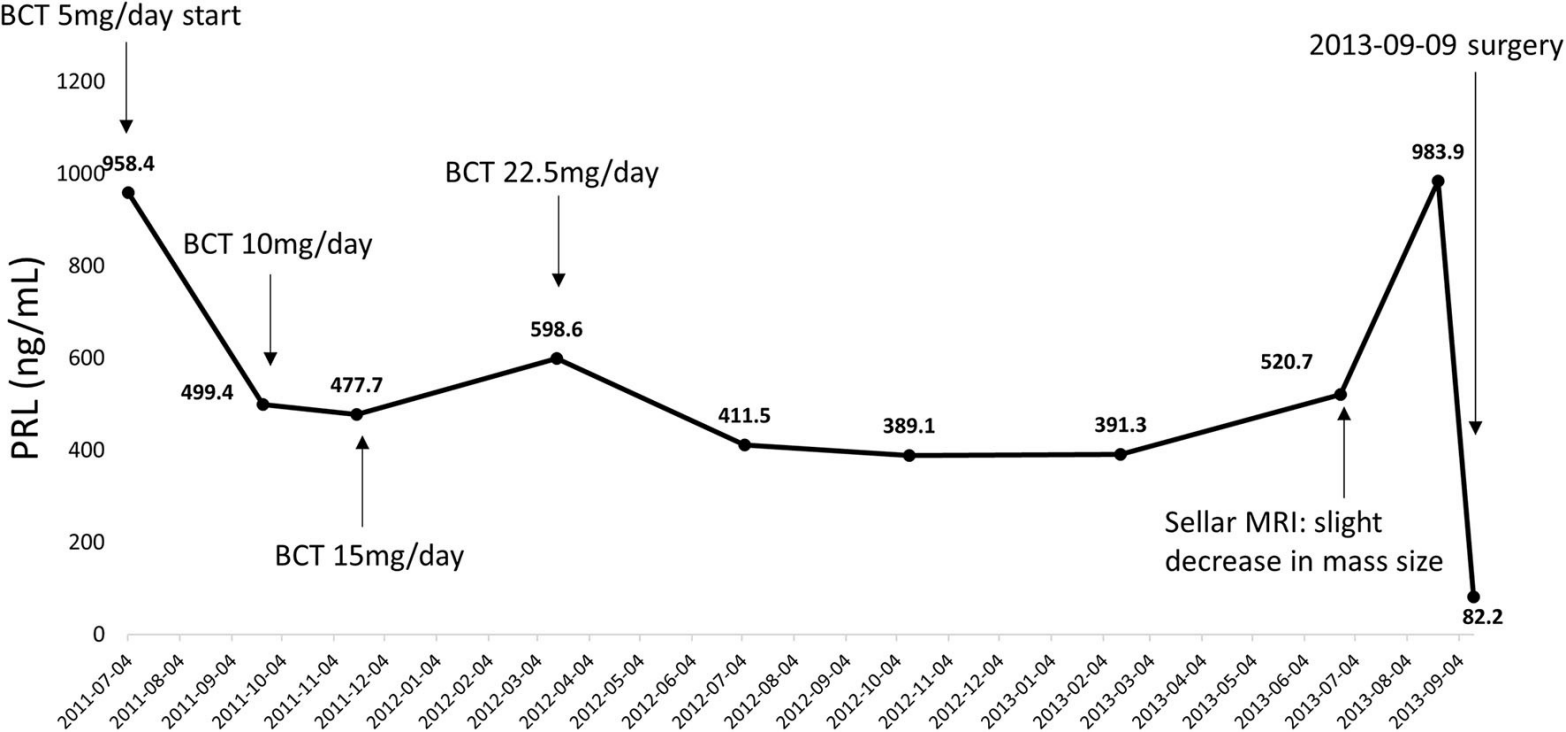
Serial changes in serum PRL levels from initial diagnosis to transsphenoidal surgery. PRL, prolactin; BCT, bromocriptine.

In 2011, a 54–years–old woman was admitted to the Department of Neurosurgery, at Chonnam National University Hwasun Hospital of South Korea. She presented with acute dizziness and visual disturbance. In 2003, the patient was diagnosed with a 1.3 cm intrasellar mass on brain computed tomography (CT) when she visited the emergency room because of headache, nausea, and vomiting. Further evaluation was not performed at that time due to loss of follow-up. Sella magnetic resonance imaging (MRI) revealed an ~1.8–cm sized pituitary mass with right cavernous sinus invasion. Serum PRL level was 958 ng/ml (reference range 0–25 ng/ml). DA therapy with bromocriptine was initiated for the treatment of macroprolactinoma. Since serum PRL levels continued to increase despite the escalation of bromocriptine doses, she underwent transsphenoidal surgery 2 years after. Prednisolone and levothyroxine was administered for postoperative panhypopituitarism, and DA was changed to cabergoline after surgery (Figure 1).

A histopathological examination showed strong positive immunohistochemical staining for PRL. The Ki-67 index was increased (6%), mitotic index was not increased (2 per 10 high-power fields [HPF] at x400 magnification), and p53 positivity was negative (4 strongly positive nuclei/10 HPF at x400 magnification).

The serum PRL level did not normalize after surgery; however, it reduced to <200 ng/ml in 2 years with cabergoline. Subsequently, in 2016, the PRL level abruptly increased to above 900 ng/ml despite the increase in cabergoline dose to 2 mg per week. A MRI scan revealed no recurrence of the pituitary mass. Considering that she was a postmenopausal woman, cabergoline therapy was temporarily suspended for a year, but was resumed because serum PRL levels reached 8,000 ng/ml. However, because her PRL levels did not decrease despite administration of cabergoline up to 3 mg per week for 6 months, cabergoline was discontinued.

**Figure 2:**
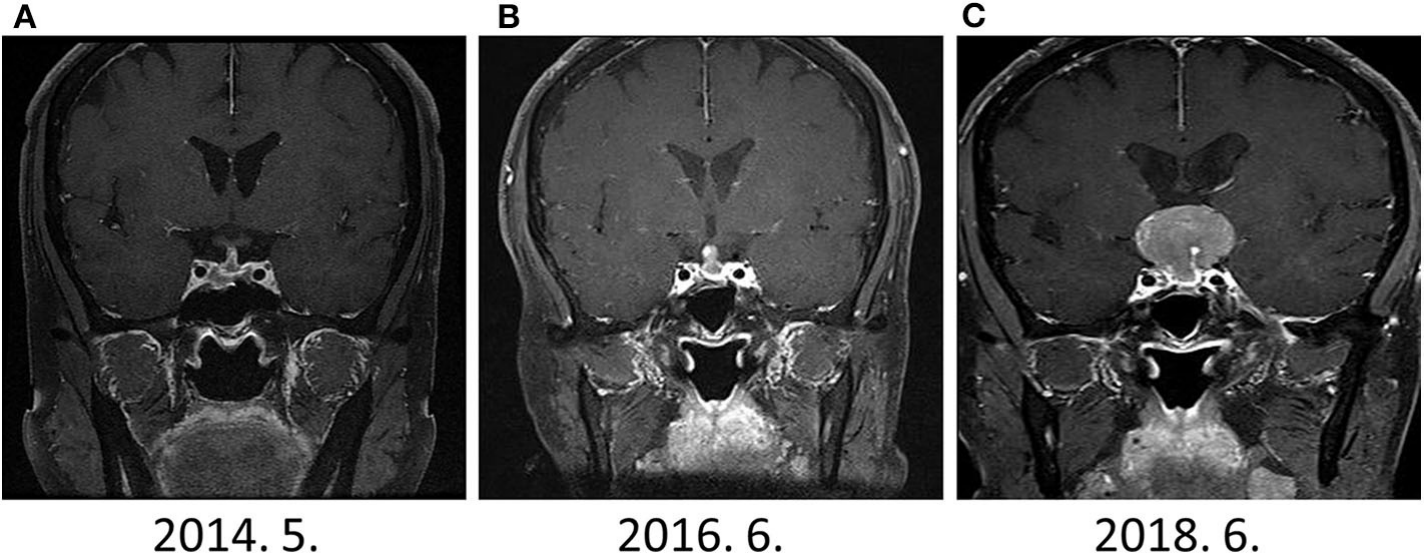
The change in sellar mass **(A)** 1-year after surgery **(B)** 3-years after surgery **(C)** 5-years after surgery. Although dramatic elevation of serum PRL levels were observed since Jun 2016, prominent sellar mass was detected on June 2018.

**Figure 3:**
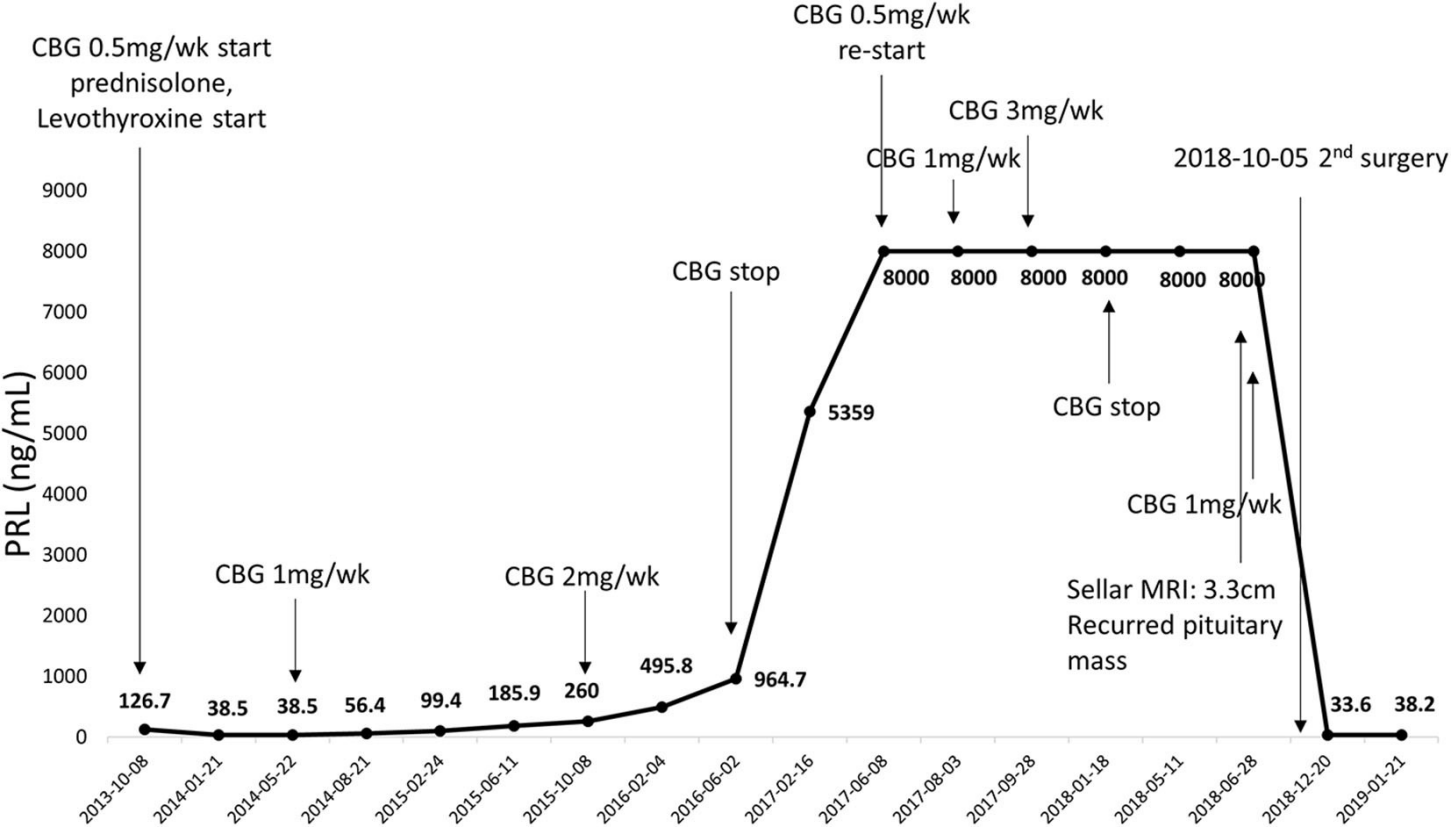
Serial changes in serum PRL levels from first surgery to second surgery. CBG, cabergoline.

During the drug holiday, in 2018, she was newly diagnosed with GIST with liver and peritoneal metastases through routine health examination. She started to receive imatinib (glivec®) for GIST. Two–months after the imatinib treatment, she suddenly presented with headache and acute visual impairment. Sella MRI showed a 3.3–cm sized recurrent pituitary mass (Figure 2). Cabergoline treatment was reinitiated, and she underwent a second transsphenoidal surgery. In addition, she received radiation therapy for a remnant tumor in the pituitary stalk. Since serum PRL levels decreased to 33 ng/ml, she did not receive DA after the second surgery (Figure 3). A histopathological examination of tissue specimen from the second surgery showed increased Ki-67 (8%) and mitotic index (3/10 HPF) indicating aggressive features of the tumor.

**Figure 4:**
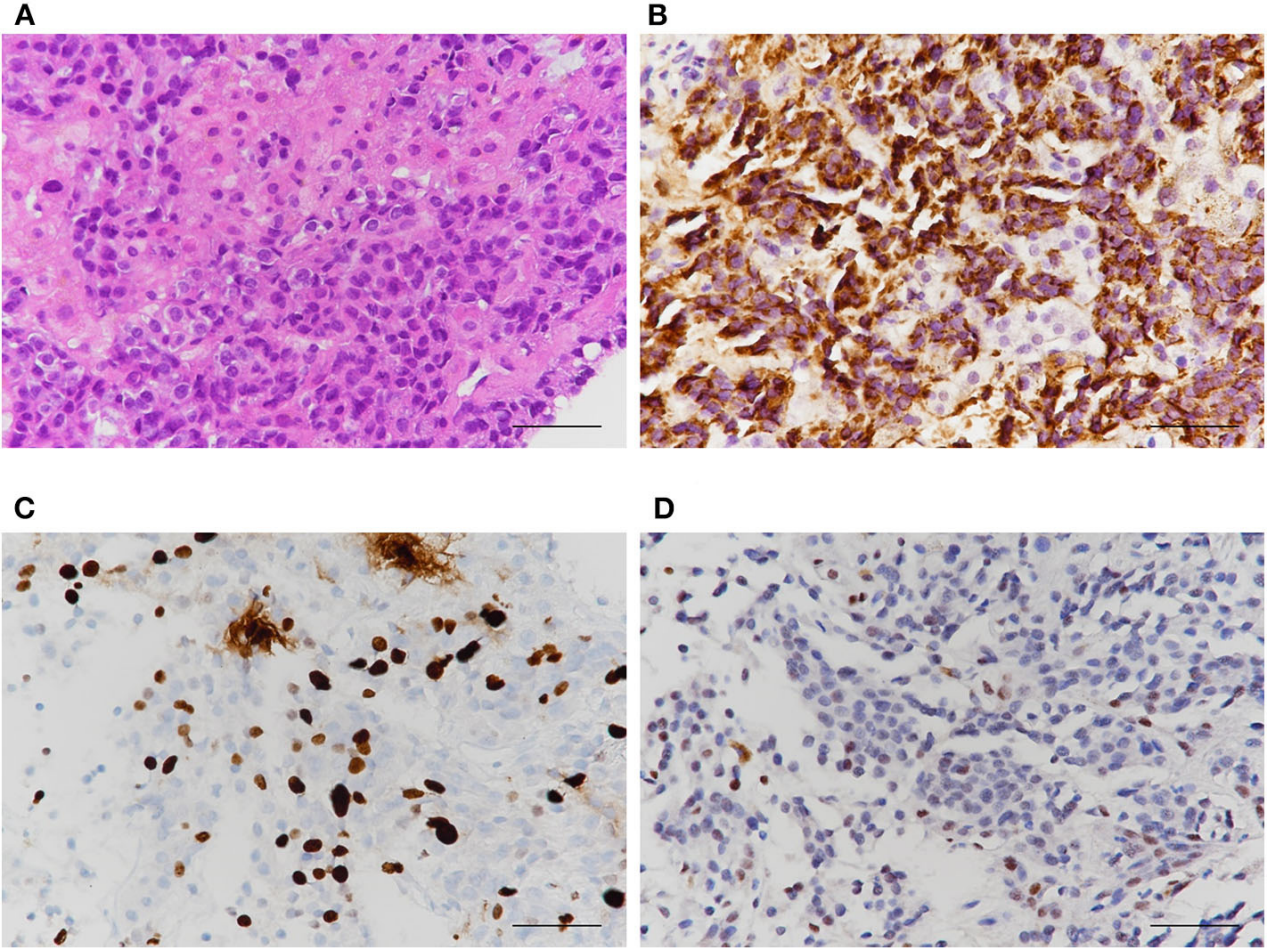
Histological characteristics of liver masses. Results of **(A)** Hematoxylin and eosin **(B)** PRL **(C)** Ki-67 index **(D)** p53 stain. PRL was strongly positive in liver masses indicating metastases from PRL-producing pituitary carcinoma, not from GIST.

With the continuation of imatinib for GIST, main mass and peritoneal metastases were dramatically decreased, whereas the size and number of liver metastases increased. Percutaneous biopsy showed that the liver masses were metastatic lesions from malignant prolactinoma and not from GIST (Figure 4). Although we considered temozolomide (TMZ) treatment, she died due to acute renal failure and sepsis 6 months after the second surgery.

## Discussion

Pituitary tumors are considered benign, but heterogeneous lesions in terms of clinical behavior. Approximately 30–45% of them are invasive, and up to 15% are regarded as clinically aggressive (4, 5). According to the recent guidelines of European Society of Endocrinology (ESE), aggressiveness is defined as a radiologically invasive tumor with an unusual rapid tumor growth or clinically relevant tumor growth despite optimal treatments (6). Pituitary carcinomas are rare diseases that can be defined only on the basis of evidence of intra- or extracranial metastasis (7). The definition cannot represent the malignant potential of the tumor that could develop metastases at a later stage of the clinical course. However, from a clinical perspective, pituitary carcinomas inevitably include aggressive features of pituitary adenoma that show a rapid growth or local invasion.

Invasiveness is radiologically or histologically determined, for example, the invasion of the cavernous sinus and the sphenoidal sinus. Histologically, aggressiveness is defined as the presence of at least two out of three proliferative markers as following: (1) Ki-67 index >1%, (2) mitotic index >2 per 10 HPF at x400 magnification, and (3) positive p53 nuclear detection (10 strongly positive nuclei/10 HPF x400 magnification) (6). In addition to increased proliferation, cellular abnormalities and vascular invasion are seen in aggressive pituitary tumors (8). Since the histological characteristics are similar between pituitary carcinomas and aggressive adenoma, it is difficult to distinguish pituitary carcinomas from aggressive pituitary adenomas based on clinical and histological aspects.

The pathogenesis of pituitary carcinomas is not fully elucidated yet. Majority of cases occur after the initial diagnosis of pituitary tumor, and there is a time delay between the initial diagnosis and the detection of metastases, with an average of 4.7 years (2 months−22 years) (2). Thus, malignant transformation from preexisting benign adenomas and not *de novo* tumors seems to be the main mechanism for the development of pituitary carcinomas. Moreover, there is a certain possibility of metastatic spread after therapeutic interventions including surgery and/or radiotherapy such as blood-borne dissemination, drop metastasis (occurrence in surgical tract), or central venous dissemination (9).

Prolactinomas comprise the most common pituitary tumor accounting for ~40% of all functional pituitary tumors (10). Among extremely rare pituitary carcinomas, PRL-producing pituitary carcinomas are the most common subtypes following ACTH-producing tumors (11). Malignant prolactinomas are more frequently found in middle-aged men than in other age groups (12). The preferential site for metastasis remains uncertain, but more than half of malignant prolactinomas show systemic metastasis rather than central nervous system metastasis (2). To the best of our knowledge, this is the first report of liver metastases in cases of malignant prolactinomas. Recently developed ^68^Ga-DOTATATE positron emission tomography (PET)-CT shows higher resolution than ^18^F-fluorodeoxyglucose (FDG) PET-CT or regional MRI, and currently, it plays a major role in the diagnosis of neuroendocrine tumors including pheochromocytoma. Considering the usefulness of ^18^F-FDG PET-CT as an indicator for tumor aggressiveness, the combination of ^68^Ga-DOTATATE PET-CT and ^18^F-FDG PET-CT may be beneficial in improving diagnostic accuracy of malignant prolactinomas.

For diagnosing malignant prolactinoma, the most crucial point is clinical suspicion for the development of metastatic lesions. Due to a very low incidence of the disease, it can be easily overlooked. In our case, despite a significant rise in PRL levels initially to 900 ng/mL, her MRI did not show any significant change. The discrepancy between the MRI and significantly elevated PRL levels should have prompted an investigation for metastatic lesions. In our case, metastatic lesions were incidentally detected during the evaluation of GIST ~2.5 years after the onset of significant rise in PRL levels.

The prognosis of malignant prolactinomas is extremely poor, with the median survival duration of 10 months after the detection of metastases (13). This means that there are no optimal treatment strategies for malignant prolactinomas, and the treatments are generally palliative. Surgery and radiotherapy are commonly considered adjuvant therapies for local disease control. Park et al. reported a case of malignant prolactinoma with fourth ventricle metastasis that was treated with Gamma-knife radiosurgery, which led to remarkably prolonged survival of 3 years (14). However, the effect of surgery and/or radiotherapy on tumor behavior and overall survival is usually limited (15).

Medical treatment with DA plays a role as first-line therapy of PRL-producing pituitary carcinomas to reduce the tumor volume. However, in case of the development of resistance to DA or evidence of metastases, DA only remains a palliative option. Divergent regimens of cytotoxic chemotherapy have been challenged, but they did not show relevant survival gain. TMZ, an oral alkylating agent, is the most promising adjuvant therapy for aggressive prolactinomas. TMZ causes alkylation of guanine to O^6^-methylguanine which promotes DNA damage by base mismatch repair. TMZ led to the reduction of tumor volume up to 47% with disease control rate of 80% in previous studies (13, 16). The ESE guidelines recommend TMZ as the first-line chemotherapy for uncontrolled malignant prolactinomas despite of standard therapies including DA or surgery (6). In our case, we had considered adjuvant TMZ therapy after the detection of liver metastases, however, we were unable to do so because of her unexpected death. Currently, there is no solid evidence to support the efficacy of other medical options after the treatment failure to TMZ. However, combination of lapatinib and cabergoline, multi-receptor-targeted somatostatin receptor ligand pasireotide, and mammalian target of rapamycin inhibitor everolimus are recently suggested as promising agents under clinical investigation (17–19).

Interestingly, our case showed the progressive liver metastases from malignant prolactinoma after initiating imatinib. There may be a possible question for the effect of imatinib on the progression of PRL-producing carcinoma. However, based on the mechanism of molecular action, the bcr-abl tyrosine kinase inhibitor imatinib affects Erk and PI3k/Akt pathway, that is a similar way of emerging therapeutics for malignant prolactinomas, lapatinib, and everolimus. Moreover, serum PRL levels were already significantly higher than 8,000 ng/ml before initiating imatinib for 2 years. Therefore, imatinib treatment seems not to be associated with the poor prognosis of our case.

Peptide receptor radionuclide therapy (PRRT) is another challenging therapy for non-medical management of PRL-producing carcinomas. PRRT basically uses the binding affinity of radiolabeled peptide analogs to peptide receptors in tumor cells (20). There are two rational candidates for the treatment of malignant prolactinomas, ^177^Lu-DOTATATE and ^90^Y-DOTATOC, which are the most frequently used radiopharmaceuticals for PRRT (21). Recent studies showed remarkable tumor shrinkages in aggressive pituitary tumors with PRRT, which also might be a helpful therapeutic option for malignant prolactinomas (22–24).

In conclusion, we presented a case of malignant prolactinoma with liver metastases, which were clinically considered as metastases of GIST but pathologically confirmed as metastases of PRL-producing carcinoma. In this case, metastases were identified incidentally during the evaluation of imatinib's response to GIST. Our case suggests that highly sustained serum PRL levels despite DA therapy may not indicate DA-resistant prolactinomas but rather malignant prolactinomas. Therefore, clinical suspicion is crucial to detect hidden metastases.

## Ethics Statement

The studies involving human participants were reviewed and approved by the Institutional Review Board of Chonnam National University Hwasun Hospital. The patients/participants provided their written informed consent to participate in this study.

## Author Contributions

AH and HK managed the case. AH drafted the manuscript. AH, JY, HK, and H-CK reviewed the manuscript. All the authors agreed with the final version of the manuscript.

## Conflict of Interest

The authors declare that the research was conducted in the absence of any commercial or financial relationships that could be construed as a potential conflict of interest.
